# Untargeted Metabolomic Analysis Using UPLC–MS/MS Reveals Metabolic Changes Associated With *Lanmaoa asiatica* Poisoning

**DOI:** 10.1002/fsn3.70583

**Published:** 2025-07-08

**Authors:** Ruanxian Dai, Zhantao Duan, Bin Han, Yating Peng, Lan Zhu, Yuan Shen, Qiang Meng

**Affiliations:** ^1^ Faculty of Life Science and Technology Kunming University of Science and Technology Kunming China; ^2^ The First People's Hospital of Yunnan Province Kunming China; ^3^ The First Affiliated Hospital of Kunming Medical University Kunming China

**Keywords:** *Lanmaoa asiatica*, metabolomics, mushroom poisoning, neuropsychiatric symptoms, oxidative phosphorylation

## Abstract

*Lanmaoa asiatica* is known for its unique flavor; however, improper consumption can induce severe neuropsychiatric symptoms, including hallucinations and irritability. The underlying toxicity mechanism remains unclear, and the lack of specific antidotes poses a significant threat to patient safety. This study employed ultra‐high performance liquid chromatography–tandem mass spectrometry (UPLC‐MS/MS) to analyze the plasma metabolic profiles of *patients with Lanmaoa asiatica* poisoning and healthy controls. A total of 20 patients were included, with an average age of 36.9 ± 13.08 years. No significant differences were observed in age, gender, or laboratory indicators between the patient and control groups (*p* > 0.05). Poisoned patients primarily exhibited neuropsychiatric symptoms, including hallucinations (75%) and general weakness (60%), along with gastrointestinal symptoms such as nausea (60%) and vomiting (45%). Metabolomic analysis identified 914 differential metabolites, primarily involving benzene derivatives, organic acids and their derivatives, amino acid metabolites, and heterocyclic compounds. Notably, 5‐methoxytryptophan (5‐MTP) and protocatechuic acid were significantly upregulated, suggesting potential pharmacological relevance. KEGG pathway analysis revealed disturbances in oxidative phosphorylation and the morphine addiction pathway, implicating mitochondrial dysfunction as a key factor in Lanmaoa asiatica toxicity. Additionally, adenosine monophosphate (AUC = 0.917), adenosine 5′‐diphosphate (AUC = 0.935), and adenosine 5′‐triphosphate (AUC = 0.895) were identified as potential metabolic biomarkers and therapeutic targets. Despite the overall favorable prognosis and no significant damage to vital organs such as the liver and kidneys, the severe hallucinogenic effects raise concerns about increased risks of self‐harm and accidental injury. However, this study has certain limitations, including a relatively small sample size and potential challenges in metabolite identification inherent to untargeted metabolomics. These factors may affect the generalizability and biological interpretation of the findings. Future studies with larger cohorts and integrated, targeted approaches are warranted to validate and refine these results.

## Graphical abstract

*Lanmaoa asiatica* possesses notable nutritional and pharmacological value, yet its potential to cause neuropsychiatric symptoms remains unclear. This study represents the first application of UPLC‐MS/MS non‐targeted metabolomics technology to systematically analyze the plasma metabolic profiles of patients with *Lanmaoa asiatica* poisoning, particularly involving mitochondrial dysfunction and morphine addiction pathways. Key metabolites such as 5‐methoxytryptophan and adenosine derivatives may be identified as potential biomarkers. However, the small sample size and limitations of untargeted metabolomics may affect the generalizability and interpretation of the results.

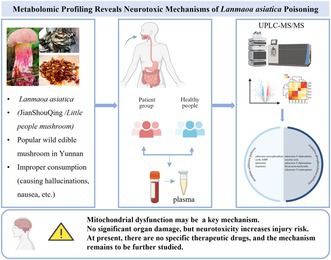

## 1 Introduction

Mushroom poisoning is reported annually worldwide (Moss and Hendrickson 2019; Somrithipol et al. 2022) and remains a significant public health issue. In southwestern China, particularly in Yunnan (Li et al. 2021, 2022), where wild edible mushrooms are abundant and residents have a longstanding dietary tradition of consuming them, mushroom poisoning frequently occurs, posing a significant threat to public health (Cheng et al. 2023).

*Lanmaoa asiatica* belongs to the family Boletaceae, Boletus, and is commonly known as “HongCong” because of the brick‐red color of the cap and the base of the stipe, and is also known as “JianShouQing” because of the blue‐black color of the damaged part of the stipe (Liu et al. 2023). Residents in Yunnan favor this fungus for its distinctive flavor. However, several case reports have indicated that improper cooking of *Lanmaoa asiatica* can lead to poisoning, with neuropsychiatric symptoms being the primary clinical manifestations. These symptoms include hallucinations, delirium, and other manifestations, and some patients may also experience gastrointestinal symptoms such as nausea, vomiting, and diarrhea (Li, Li, et al. 2023; Dai et al. 2024). Due to the prevalence of reports from poisoned individuals describing visions of “little people” at the onset of their symptoms, this fungus is colloquially referred to as “little people mushroom” in local folklore. Currently, the treatment of poisoning primarily relies on non‐specific supportive therapy, and there is a notable absence of targeted antidotes.

Additionally, psychiatric symptoms associated with poisoning elevate the risk of accidents. Thirty‐two toxic mushrooms have been identified as capable of inducing neuropsychiatric symptoms, with *Lanmaoa asiatica* being the most significant (Li, Lockwood, et al. 2023). However, current studies on this mushroom predominantly focus on its flavor, nutrient composition, and pharmacological activity (Su et al. 2018; Yang et al. 2022; Tang et al. 2024; Wang et al. 2024), while research on the mechanisms underlying its toxicity remains relatively scarce, with most available literature consisting of case reports. Consequently, analyzing the effects of *Lanmaoa asiatica* poisoning on body metabolism is crucial for elucidating the mechanisms of its toxic effects.

Metabolomics has become a widely utilized approach in toxicology research, offering innovative insights into the mechanisms of toxicity and facilitating early diagnosis (Zheng et al. 2023). However, there is currently no metabolomic investigation concerning the toxicity of *Lanmaoa asiatica*. In this study, we employed untargeted metabolomics technology using ultra‐performance liquid chromatography–tandem mass spectrometry (UPLC–MS/MS) to analyze plasma samples collected from patients *who had been poisoned by Lanmaoa asiatica*. Our systematic analysis of their metabolic profiles aims to identify potential biomarkers and elucidate the underlying mechanisms of toxicity. This research will contribute a novel scientific foundation for the toxicological investigation of *Lanmaoa asiatica* poisoning.

## 2 Materials and Methods

### 2.1 Object of Study

**FIGURE 1:**
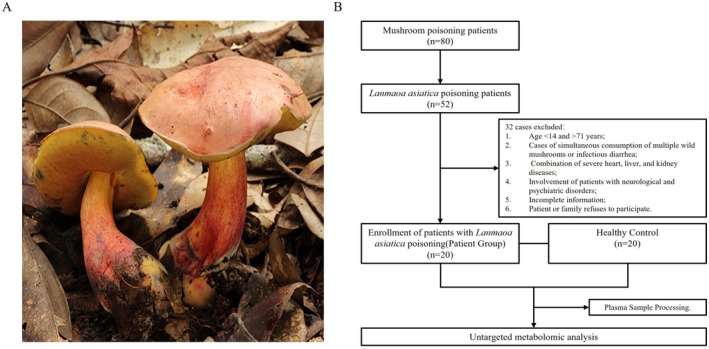
(A) *Lanmaoa asiatica*. (B) Experimental design.

**FIGURE 2:**
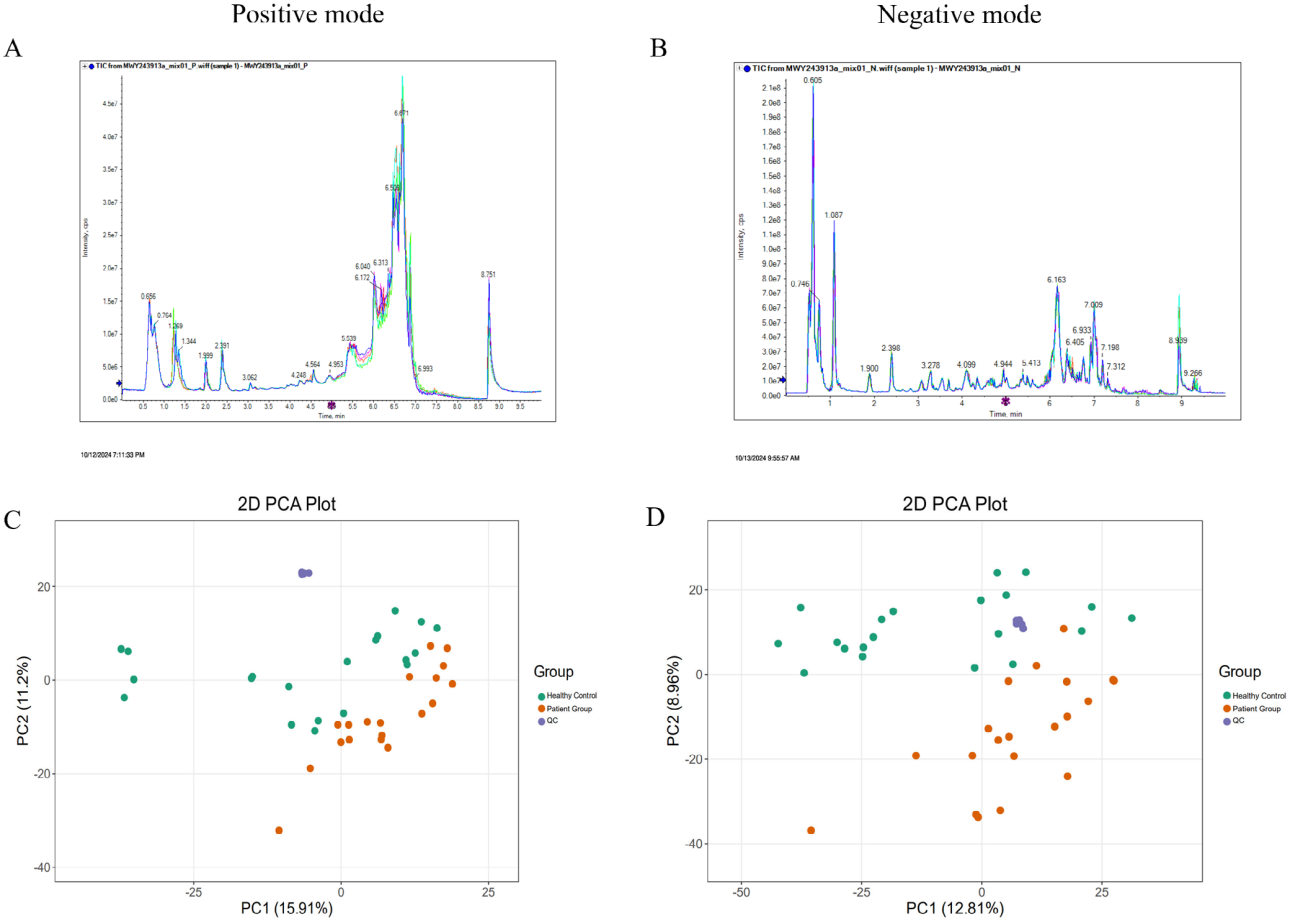
(A) Total ion chromatogram in positive ion mode. (B) Total ion chromatogram in negative ion mode. (C) The Principal component analysis (PCA) of QC samples in positive ion mode. (D) The Principal component analysis (PCA) of QC samples in negative ion mode.

In this study, we collected data from a total of 20 patients diagnosed with *Lanmaoa asiatica* poisoning in the Department of Emergency Medicine at the First People's Hospital of Yunnan Province between June 2024 and August 2024, constituting the poisoning group. Simultaneously, 20 healthy volunteers were recruited during the same period to serve as the normal control group. The study received approval from the Medical Ethics Committee of the First People's Hospital of Yunnan Province (Approval No. KHLL2023‐KY064). It adhered strictly to the ethical principles outlined in the Declaration of Helsinki. All tests were conducted after obtaining informed consent from the subjects or their legal guardians. The inclusion criteria for the *Lanmaoa asiatica* poisoning group were as follows: (1) clear identification of *Lanmaoa asiatica* ingestion confirmed by fungal mapping (Figure 1A); (2) an age range of 15 to 70 years; and (3) onset of poisoning within 24 h. The exclusion criteria included: (1) individuals who consumed multiple wild mushrooms or experienced concurrent infectious diarrhea; (2) those with severe cardiac, hepatic, or renal diseases; (3) individuals with pre‐existing neurological or psychiatric disorders; (4) subjects with incomplete clinical data; and (5) individuals who declined participation in the study, either personally or through their family members. The experimental design is illustrated in Figure 1B.

### 2.2 Sample Preparation and Extraction

Blood was collected from the subjects intravenously using vacuum blood collection tubes (BD Vacutainer) containing EDTA‐K2 anticoagulant. Immediately after collection, the plasma was gently mixed and separated by centrifugation at 4000 rpm for 10 min at 4°C. The separated plasma was then dispensed into pre‐cooled EP tubes, quickly frozen in liquid nitrogen, and stored at −80°C until analysis.

The plasma sample stored at −80°C in the refrigerator was thawed on ice and vortexed for 10 s. Fifty microlitre of sample and 300 μL of extraction solution (ACN: Methanol = 1:4, V/V, This combination is widely utilized in non‐targeted metabolomics and is particularly effective for extracting both polar and moderately polar small molecule metabolites.) (Zheng et al. 2020) containing internal standards were added into a 2 mL microcentrifuge tube. In the positive mode, the internal standards utilized were caffeine‐^13^C_3_ (0.2 mg/L), L‐Leucine‐D7 (1 g/L), L‐Tryptophan‐D5 (0.5 mg/L), and 2‐Amino‐3‐(2‐Chlorophenyl)Propanoic Acid (0.2 mg/L). Conversely, in the negative mode, the internal standards employed were benzoic acid‐D5 (1 mg/L), L‐Tryptophan‐D5 (0.5 mg/L), Hexanoic Acid‐D11 (1 mg/L), and 2‐amino‐3‐(2‐chlorophenyl)Propanoic Acid (0.2 mg/L). The internal standards were incorporated into the extraction solvent primarily to monitor the stability of the instrument throughout the detection process. The sample was vortexed for 3 min and then centrifuged at 12,000 rpm for 10 min (4°C). 200 μL of the supernatant was collected and placed at −20°C for 30 min and then centrifuged at 12,000 rpm for 3 min (4°C). A 180 μL aliquot of supernatant was transferred for LC‐MS analysis.

### 2.3 Untargeted Metabolomics

Chromatographic separations were performed using a Shimadzu LC‐30A UHPLC system. All samples were for two LC/MS methods. One aliquot was analyzed using positive ion conditions. It was eluted from the T3 column (Waters ACQUITY Premier HSS T3 Column 1.8 μm, 2.1 mm × 100 mm) using 0.1% formic acid in water as solvent A and 0.1% formic acid in acetonitrile as solvent B in the following gradient: 5 to 20% in 2 min, increased to 60% in the following 3 min, increased to 99% in 1 min and held for 1.5 min, then came back to 5% mobile phase B within 0.1 min, held for 2.4 min. The analytical conditions were as follows: column temperature, 40°C; flow rate, 0.4 mL/min; injection volume, 4 μL. Another aliquot was prepared under negative ion conditions and had the same elution gradient as the positive mode.

Mass spectrometry analysis was performed using a SCIEX TripleTOF 6600+ mass spectrometer (Foster City, CA, USA). The data acquisition was operated using the information‐dependent acquisition (IDA) mode using Analyst TF 1.7.1 Software (Sciex, Concord, ON, Canada). The source parameters were set as follows: ion source gas 1 (GAS1), 50 psi; ion source gas 2 (GAS2), 50 psi; curtain gas (CUR), 25 psi; temperature (TEM), 550°C; declustering potential (DP), 60 V, or‐ 60 V in positive or negative modes, respectively; and ion spray voltage floating (ISVF), 5000 V or‐ 4000 V in positive or negative modes, respectively. The TOF MS scan parameters were set as follows: mass range, 50–1000 Da; accumulation time, 200 ms; and dynamic background subtract, on. The product ion scan parameters were set as follows: mass range, 25–1000 Da; accumulation time, 40 ms; collision energy, 30 or‐ 30 V in positive or negative modes, respectively; collision energy spread, 15; resolution, UNIT; charge state, 1 to 1; intensity, 100 cps; exclude isotopes within 4 Da; mass tolerance, 50 ppm; maximum number of candidate ions to monitor per cycle, 18.

### 2.4 Data Processing

The raw data were converted to the mzXML format using ProteoWizard and processed with the R package XCMS (version 3.2). Peak areas were normalized using the SVR method. In the process of metabolite annotation, we comprehensively utilized multi‐dimensional information, including primary MS1 information (parent ion m/z), secondary MS2 fragment ion spectra, and retention time (RT). The specific annotation strategies are as follows: peak matching criteria were established with an m/z error of ≤ 25 ppm and an RT error of ≤ 6 s for matching the primary parent ion (Q1) and the secondary fragment ion spectra. For database search parameters, the MS1 search error was set to ≤ 25 ppm, the MS2 fragment matching error was set to ≤ 50 ppm, and the RT matching error was set to ≤ 60 s. The MS2 spectral searching was conducted using a comprehensive scoring system, with a minimum matching score threshold set at 0.3. The final score was composed of three components: Fragment score (10% weighting), Forward Score (30% weighting), and Reverse Score (60% weighting).

Following peak correcting and filtering, metabolite identification was performed by querying the laboratory's in‐house database (MetWare Biological Science and Technology Co. Ltd., Wuhan, China), which comprises over 2000 reference specimens. This database was constructed using purchased analytical standards and data acquired under non‐targeted chromatography‐mass spectrometry (NTSC‐MS) conditions. In our study, we integrated several prominent international public databases, including METLIN (http://metlin.scripps.edu), HMDB 4.0 (https://hmdb.ca), KEGG (https://www.kegg.jp), MoNA (https://mona.fiehnlab.ucdavis.edu), and MassBank (http://www.massbank.jp). Additionally, we employed the MetDNA approach and AI algorithm‐based prediction libraries to further assist in structural inference and annotation. We assessed the metabolite annotation results using a confidence level based on the guidelines of the Metabolomics Standards Initiative (MSI).

After preprocessing, the data was analyzed to visualize the metabolic profiles through multivariate statistical techniques, including principal component analysis (PCA), hierarchical cluster analysis, orthogonal partial least squares discrimination analysis (OPLS‐DA), and Student's *t*‐test. Differential metabolites were selected based on the combined thresholds of Student's *t*‐test (*p* < 0.05) and variable importance in projection (VIP > 1) from the OPLS‐DA model. Pathway annotation of differential metabolites in brain tissues was performed using the Kyoto Encyclopedia of Genes and Genomes (KEGG) database (http://www.kegg.com/).

In Principal Component Analysis (PCA) and heat map analysis, we employed the UV standardization method (Unit Variance Scaling), which involves mean centering each variable and dividing by the standard deviation to ensure uniform variance across all variables. This approach effectively mitigates the influence of variable magnitude on the model results. In the OPLS‐DA analysis, we implemented log2 transformation followed by post‐centering (Zero‐centered) to enhance the explanatory power and stability of the model. Specifically, we first log‐transformed the variables to address the impact of skewed distributions, followed by mean centering to eliminate systematic bias among the samples. All of the aforementioned preprocessing methods adhere to standard procedures for routine metabolomics data analysis and have been extensively utilized in several related studies. Metabolomic sequencing was conducted by Wuhan Metwell Biotechnology Co. Ltd. (Wuhan, China).

### 2.5 Statistical Analysis

All statistical analyses were conducted using SPSS version 26. The Shapiro–Wilk test was employed to assess the normality of the data. Based on the results of this test, the unpaired *t*‐test was used for comparisons between two groups when the data followed a normal distribution. Conversely, the Mann–Whitney rank sum test was applied for comparisons when the data did not conform to a normal distribution. Continuous data are presented as means and standard deviations, categorical data as frequencies and percentages, and non‐normally distributed results as medians with the 25th and 75th percentiles (*M* [P25, P75]). *p* < 0.05 was considered the level of significance.

ROC curve analysis was employed to evaluate the capacity of candidate differential metabolites to distinguish between subgroups. This analysis utilized the complete dataset without partitioning it into training and test sets or employing cross‐validation methods (e.g., MCCV). The results were intended solely to aid in assessing the discriminative efficacy of the metabolites rather than for evaluating model prediction performance. In this study, both the control and experimental groups consisted of 20 samples each, ensuring balanced sample representation during the prediction process and thereby eliminating any sample imbalance that could skew the accuracy and other indices. The area under the curve (AUC) was used to assess the predictive ability of differential metabolites about mushroom poisoning, with an AUC greater than 0.80 indicating strong discriminatory power.

## 3 Results

### 3.1 Clinical Characteristics of Patients With *Lanmaoa asiatica* Poisoning

This study included a total of 20 patients diagnosed with *Lanmaoa asiatica* poisoning, all of whom received symptomatic treatments, including hydration, diuresis, and maintenance of electrolyte balance. Additionally, sedation was administered to those exhibiting neuropsychiatric symptoms. After 1 month of follow‐up, all patients were clinically cured. The basic characteristics and laboratory test results of both the patient group and the healthy control group are detailed in Table 1, revealing no significant differences between the two groups in terms of age, gender, and laboratory indicators. The primary clinical manifestations observed in the poisoned patients included neuropsychiatric and gastrointestinal symptoms. Neuropsychiatric symptoms predominantly presented as hallucinations and generalized weakness, while gastrointestinal symptoms primarily manifested as nausea and vomiting. Detailed results are presented in Table 2.

**TABLE 1:** Baseline characteristics.

Characteristics	All	Patient group	Healthy control	*p*
People	40	20	20	—
Gender	40	20	20	0.705
Age	38.70 ± 13.41	36.9 ± 13.08	40.82 ± 13.87	0.213
Incubation period (h)	—	4 (4–11.5)	—	—
Hospital stay (h)	—	24 (10–72)	—	—
WBC (×10^9^/L)	7.58 (6.07–8.92)	7.02 (5.48–8.66)	8.17 (7.05–9.07)	0.134
Hb (g/L)	138.95 ± 14.06	138.20 ± 13.86	138.82 ± 14.68	0.626
PLT (×10^9^/L)	237.88 ± 74.49	253.9 ± 68.82	219.02 ± 78.50	0.12
AST (U/L)	20 (17–23)	20 (17–23.92)	20 (16–22.5)	0.529
ALT (U/L)	19 (14.1–24.5)	18.5 (13.3–23.68)	20 (14.5–24.5)	0.94
DB (μmol/L)	2.12 (1.65–2.69)	2.22 (1.95–2.77)	1.80 (1.5–2.65)	0.445
Cre (μmol/L)	53 (49–63)	54 (47.75–63.0)	52 (49–64)	0.968
BUN (mmol/L)	4.622 ± 1.47	4.22 ± 1.12	5.1 ± 1.70	0.08
Glu (mmol/L)	5.7 (5.15–6.35)	5.55 (5.2–6.0)	5.70 (5.1–6.45)	0.968
APTT (s)	34.55 ± 2.89	34.90 ± 2.90	34.14 ± 2.91	0.826
PT (s)	13.6 (13–14)	13.45 (12.75–13.77)	13.6 (13.4–14.2)	0.149

**TABLE 2:** Clinical characteristics of *Lanmaoa asiatica* poisoning patients.

Characteristics	Number of patients (%)
Neuropsychiatric symptoms	
Hallucinations	15 (75%)
General weakness	12 (60%)
Dizziness	7 (35%)
Irritable	1 (5%)
Gastrointestinal symptoms	
Nausea	12 (60%)
Vomiting	9 (45%)
Diarrhea	4 (20%)
Abdominal pain	3 (15%)

### 3.2 Data Quality Evaluation

In this study, quality control (QC) samples were prepared by mixing equal volumes of extracts from all biological samples to evaluate the stability and reproducibility of the instrumental analysis process. During the mass spectrometry analysis, we implemented a strategy of inserting one QC sample every 10 assay samples to continuously monitor the stability of the instrumental signal and the reproducibility of the method in real time. The specific injection sequence of the samples is provided in Figure S1.

The Total Ion Chromatogram (TIC) demonstrated that the peaks of QC samples exhibited a consistent trend in both positive and negative ion modes, with good signal stability, indicating that the detection results are reliable (Figure 2A,B). Furthermore, the PCA score plot revealed that the QC samples were closely clustered, with minimal differences in both ion modes, suggesting that the methodology employed in this study possesses strong stability and reproducibility and that the data are highly reliable (Figure 2C,D).

### 3.3 Multivariate Statistical Analysis

**FIGURE 3:**
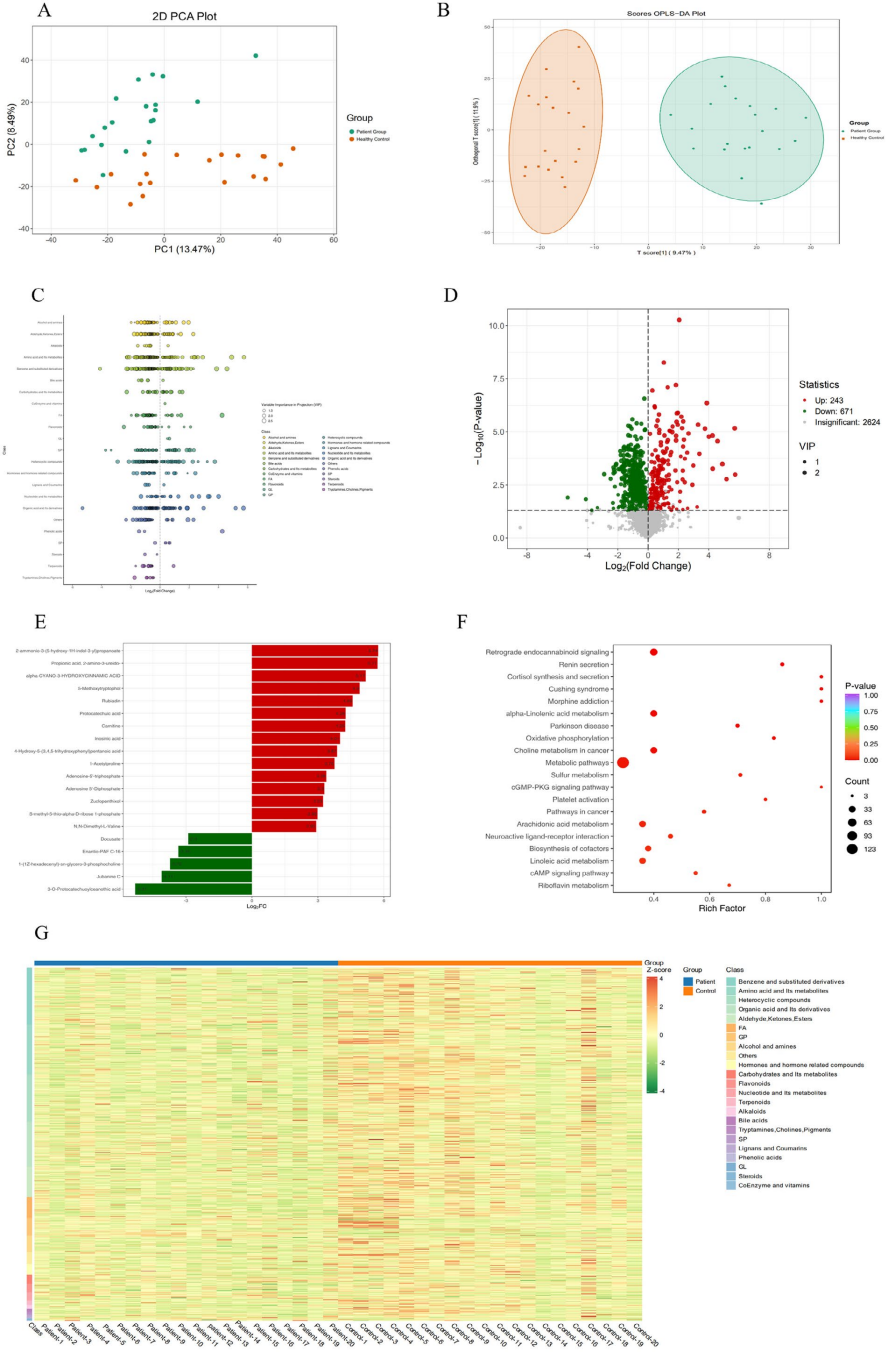
Metabolomics profiling of patient group and healthy control. (A) Principal component analysis (PCA) demonstrates distinct clustering patterns between healthy controls (red circles) and patient groups (green circles). (B) Orthogonal partial least squares‐discriminant analysis (OPLS‐DA) score plot reveals clear separation between healthy controls (red circles) and patient groups (green circles). (C) Differential metabolite distribution plot showing log2‐transformed fold change (log2FC) versus variable importance in projection (VIP) scores. The horizontal axis represents the magnitude of content difference between groups, with color coding indicating metabolite classification. (D) Volcano plot visualization of differential metabolites, highlighting significantly upregulated (red), downregulated (green), and non‐significant metabolites (gray) based on fold change and statistical thresholds. (E) Bar chart quantification of differential metabolite expression patterns (red: Upregulated; green: Downregulated). (F) Pathway enrichment analysis scatter plot displays significantly altered metabolic pathways, where circle size corresponds to the number of annotated metabolites, color intensity reflects *p*‐value significance (redder hues indicate lower *p*‐values), and horizontal position indicates enrichment factor magnitude. (G) Clustered heatmap visualization of differential metabolites across samples, with red indicating high content and green indicating low content.

Principal component analysis (PCA) of the plasma metabolites from the two groups revealed a significant differentiation between the poisoned patient group and the healthy control group in terms of their metabolic profiles (Figure 3A). This finding indicates substantial differences in plasma metabolites between the two groups. To further elucidate the metabolic discrepancies, orthogonal partial least squares discriminant analysis (OPLS‐DA) was employed to compare the samples from both groups. The OPLS‐DA score plot illustrated a clear separation of the poisoned patient group from the healthy control group, thereby reinforcing the conclusion that the plasma metabolic profiles of poisoned patients differ markedly from those of healthy controls (Figure 3B).

### 3.4 Metabolite Identification and Pathway Analysis

Based on the screening criteria of *p* < 0.05 and VIP > 1, a total of 914 differential metabolites were identified, primarily categorized into four major groups: benzene ring derivatives, organic acids and their derivatives, amino acids and their metabolites, and heterocyclic compounds (Figure 3C). Among these, 243 metabolites were significantly upregulated, while 671 metabolites were significantly down‐regulated (Figure 3D). Compared to the healthy control group, 15 of the top 20 differential metabolites were upregulated in the patient group, including 5‐methoxytryptophol (5‐MTP), protocatechuic acid, adenosine‐5′‐triphosphate, and adenosine 5′‐diphosphate, etc. Conversely, 5 metabolites were down‐regulated, such as Jubanine C and 3‐O‐Protocatechuoylceanothic acid, etc. (Figure 3E, Table S1). Furthermore, the trends of these differential metabolites were visualized using clustered heat maps, which further illustrated significant differences in metabolic levels between the patient group and the healthy control group (Figure 3G).

Based on the results of differential metabolite analysis, a subsequent KEGG pathway enrichment analysis was conducted, identifying a total of 189 metabolic pathways. Among these, the top 20 pathways with the lowest *p*‐values were selected, sorted in ascending order of P‐value, and visualized using a bubble diagram (Figure 3F). In this figure, the size of the bubbles represents the number of differential metabolites enriched, while the color shades indicate their significance levels (*p*‐values). The top 20 metabolic pathways identified include Retrograde endocannabinoid signaling, morphine addiction, alpha‐linolenic acid metabolism, Parkinson's disease, and oxidative phosphorylation, among others.

**FIGURE 4:**
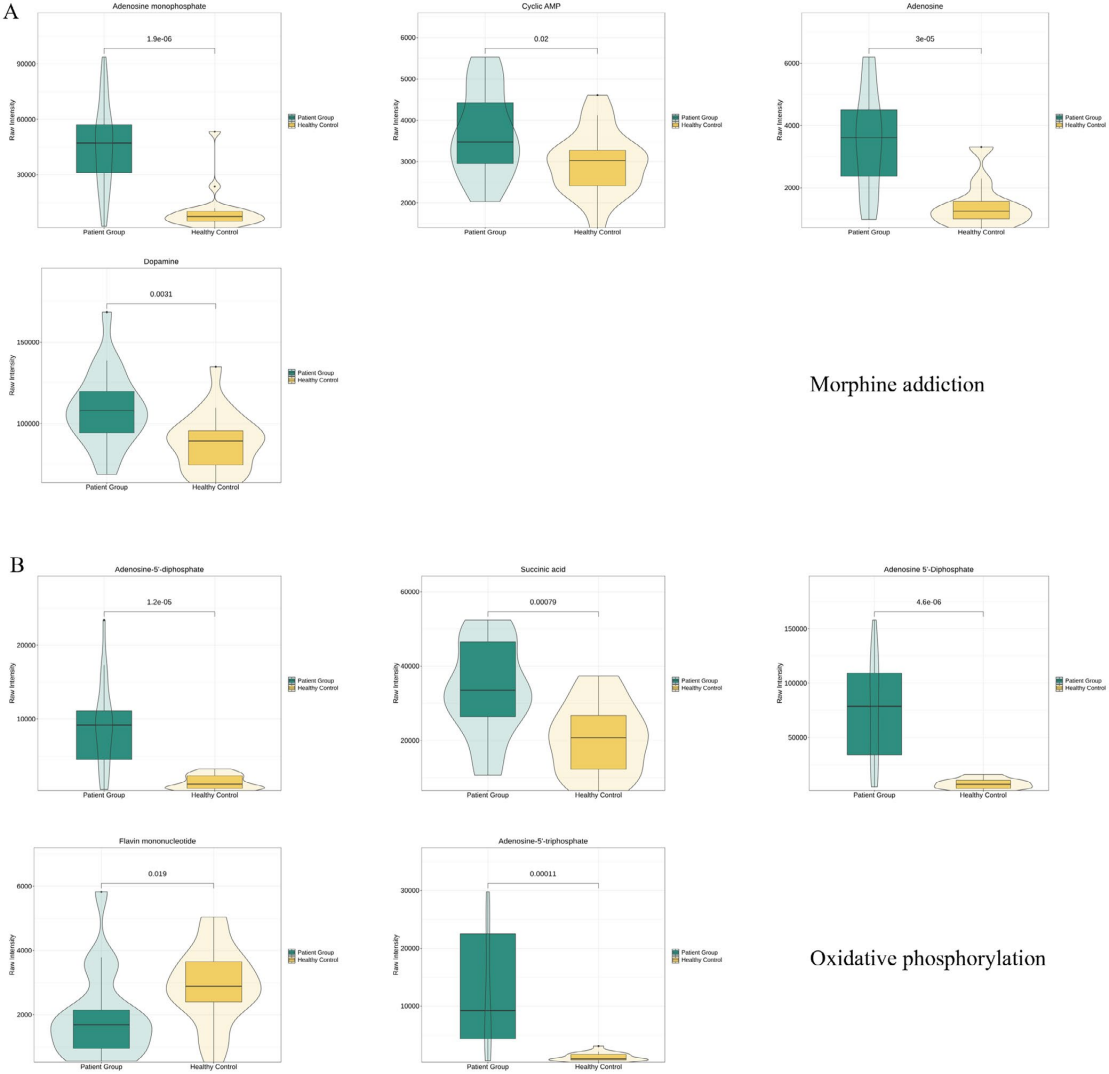
Pathway‐specific metabolite analysis. (A) Morphine addiction pathway metabolite map showing statistically significant alterations (*p* < 0.05) in the patient group versus healthy control. (B) Oxidative phosphorylation pathway metabolite map showing statistically significant alterations (*p* < 0.05) in the patient group versus healthy control.

**FIGURE 5:**
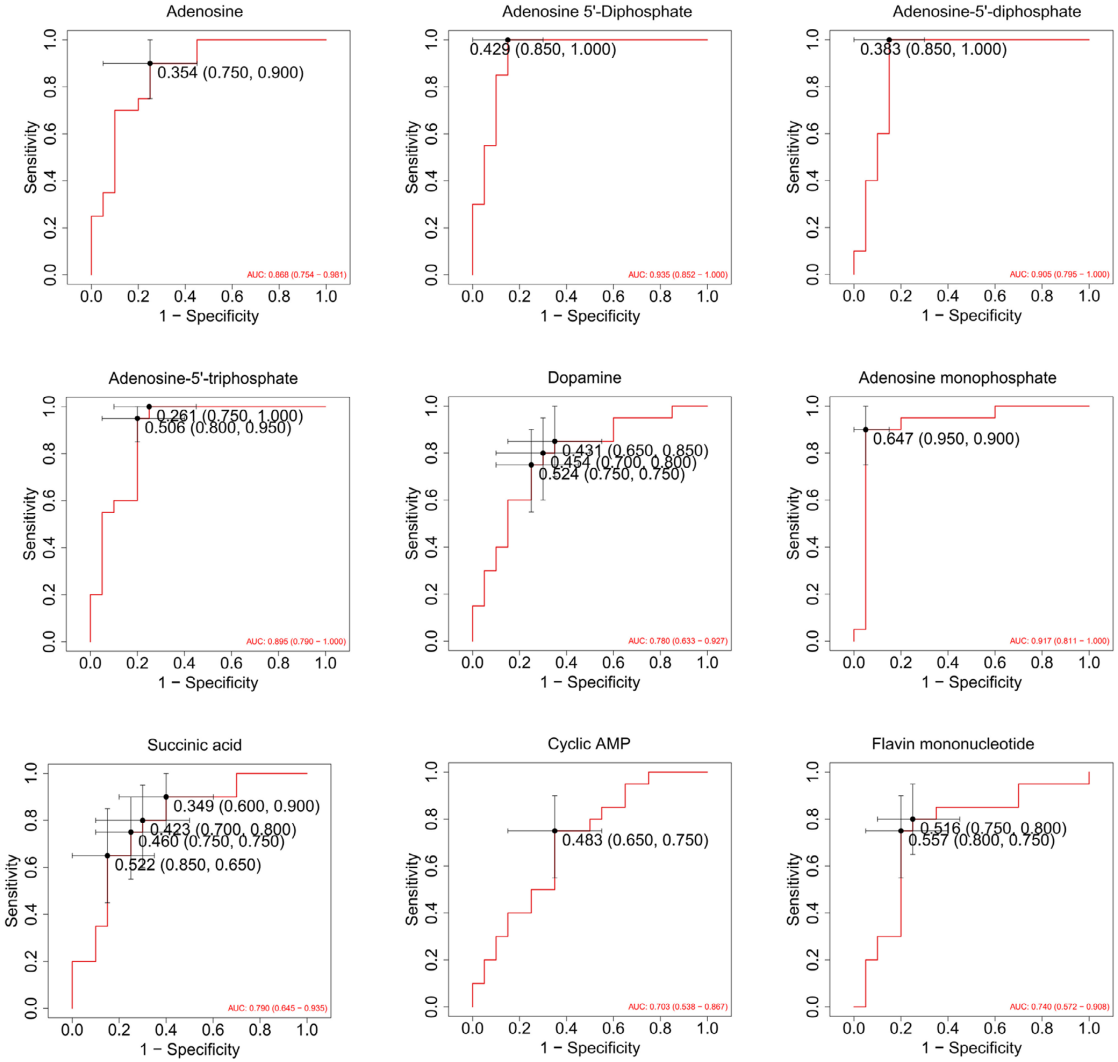
Differential metabolite ROC curve analysis of morphine addiction and oxidative phosphorylation pathways. ROC curve analysis identifies Adenosine monophosphate (AUC = 0.917), Adenosine 5′‐diphosphate (AUC = 0.935), and Adenosine‐5′‐triphosphate (AUC = 0.895) as high‐precision biomarkers for distinguishing patients from healthy controls.

Based on the results of the enrichment analysis and in conjunction with clinical symptoms, the morphine addiction pathway and the oxidative phosphorylation pathway were identified as key metabolic pathways of interest. Key metabolites enriched in the morphine addiction pathway included adenosine monophosphate, cyclic AMP, adenosine, and dopamine (Figure 4A). Key metabolites enriched in the oxidative phosphorylation pathway included adenosine‐5′‐diphosphate, succinic acid, adenosine 5′‐diphosphate, flavin mononucleotide, and adenosine‐5′‐triphosphate (Figure 4B). Quantitative analysis of differential metabolites in both pathways revealed significant differences in metabolite levels between the patient group and healthy controls. Further ROC curve analysis demonstrated high discriminatory power for the following metabolites: adenosine monophosphate (AUC = 0.917), adenosine 5′‐diphosphate (AUC = 0.935), and adenosine‐5′‐triphosphate (AUC = 0.895). These metabolite AUC values, close to or exceeding 0.9, indicate strong discriminatory power and suggest their potential as biomarkers for the discrimination or subgroup analysis of intoxicated patients (Figure 5). The analysis of these enriched pathways elucidated key metabolic abnormalities that may be implicated in the poisoning caused by *Lanmaoa asiatica*, providing significant insights for further investigation into its toxicity mechanism.

## 4 Discussion

In the present study, we systematically analyzed the plasma metabolic profiles of patients with *Lanmaoa asiatica* poisoning using an untargeted metabolomics approach for the first time. The results demonstrated that *Lanmaoa asiatica* intoxication induces significant alterations in plasma metabolites and metabolic pathways, primarily associated with four major metabolite categories: benzene ring derivatives, organic acids and their derivatives, amino acids and their metabolites, and heterocyclic compounds. These alterations may reflect the body's stress response, abnormal energy metabolism, and neurotoxicity in a state of toxicity. Enrichment analysis results, combined with clinical manifestations, further indicated that the morphine addiction pathway and the oxidative phosphorylation pathway play crucial roles in the poisoning mechanism, suggesting their involvement in toxin‐induced nerve damage and mitochondrial dysfunction. Furthermore, ROC curve analysis revealed that adenosine monophosphate (AUC = 0.917), adenosine 5′‐diphosphate (AUC = 0.935), and Adenosine‐5′‐triphosphate (AUC = 0.895) could serve as potential metabolic markers and intervention targets. This study offers new insights into the molecular mechanisms of *Lanmaoa asiatica* poisoning from a metabolomics perspective, laying the groundwork for subsequent mechanistic studies and the development of optimized clinical prevention and treatment strategies.

In this study, some of the 20 patients exhibited neuropsychiatric symptoms, while others presented with a combination of gastrointestinal symptoms. All laboratory parameters for the patients remained within the normal range, and no fatal cases were reported. Accordingly, we conclude that poisoning from *Lanmaoa asiatica* does not significantly impair the function of vital organs, such as the liver and kidneys, and the overall prognosis is favorable, aligning with findings from previous studies (Li, Peng, et al. 2023; Dai et al. 2024). However, the severe hallucinogenic effects associated with this poisoning raise concerns regarding an increased risk of self‐injury and accidents among patients, necessitating enhanced monitoring and preventive measures during clinical management. Additionally, it has been documented that hallucinogenic mushroom poisoning may induce acute renal failure in rare cases (Diaz 2021). Therefore, a heightened level of vigilance is essential when treating patients with *Lanmaoa asiatica* poisoning, and such cases should not be underestimated.

In this study, the top 20 differential metabolites were identified through LC–MS analysis, with a particular focus on 5‐MTP. 5‐MTP is an endogenous molecule derived from the catabolism of tryptophan within the tryptophan hydroxylase pathway. Our findings indicate that this metabolite is significantly upregulated in patients suffering from Lanmaoa asiatica intoxication, suggesting that Lanmaoa asiatica may disrupt central nervous system homeostasis by modulating the tryptophan metabolic pathway. Previous research has indicated that disruptions in the tryptophan‐kynurenine pathway and reduced tryptophan availability are closely linked to the emergence of neuropsychiatric symptoms (Li, Zhang, et al. 2023). Furthermore, KEGG pathway analysis revealed disturbances in the morphine addiction signaling pathway, which plays a crucial role in neuromodulation, inflammation, and immune responses; abnormalities in this pathway may exacerbate neurological damage (Kupnicka et al. 2020). These metabolic disturbances may be intricately associated with neuropsychiatric symptoms, such as hallucinations and generalized weakness, observed in patients with *Lanmaoa asiatica* intoxication, thereby providing a novel perspective for further investigation into its toxicological mechanisms.

Additionally, studies have shown that 5‐MTP plays a crucial role in regulating circadian rhythms, reproduction, and sexual function (Ouzir et al. 2013). Recent research indicates that 5‐MTP exhibits significant antifibrotic effects, effectively alleviating injury‐induced fibrosis in hepatic, renal, cardiac, and pulmonary tissues (Wu 2021). Consequently, it is regarded as a promising lead compound for the development of novel antifibrotic drugs. Furthermore, studies have shown that 5‐MTP possesses antioxidant and immunomodulatory properties (Şehirli and Sayıner 2021), along with protective effects against acute pulpitis, suggesting potential applications in the treatment of oral diseases (Kermeoğlu et al. 2021). Notably, 5‐MTP has recently been identified as a novel endothelial factor with vasoprotective and anti‐inflammatory effects. Research indicates that it not only maintains endothelial barrier function and promotes endothelial repair but also inhibits the migration and proliferation of vascular smooth muscle cells by blocking the activation of the p38 MAPK signaling pathway (Wu et al. 2020). In a mouse model, administration of 5‐MTP effectively mitigated arterial intimal hyperplasia, reduced systemic inflammation, and prevented renal fibrosis (Chou and Chan 2014).

Additionally, 5‐MTP has been shown to decrease sepsis‐related organ failure and mortality by targeting macrophages (Wang et al. 2016). The role of 5‐MTP in cancer research has also garnered significant attention, as it has been found to inhibit COX‐2 expression, thereby suppressing cancer cell migration, epithelial‐mesenchymal transition, and metastasis (Wu et al. 2014). In conclusion, 5‐MTP, a novel tryptophan metabolite, demonstrates substantial potential in anti‐inflammatory, antifibrotic, and cancer‐prevention applications, offering new therapeutic strategies for complex human inflammatory diseases.

Another metabolite of interest identified in this study is Protocatechuic Acid, which was found to be upregulated in the plasma of patients with Lanmaoa asiatica intoxication. Protocatechuic acid is a naturally occurring phenolic acid with major metabolic pathways, including glucuronidation and sulfation. This metabolite has been extensively documented to exhibit a variety of biological activities, including antioxidant, anti‐inflammatory, and neuroprotective effects (Song et al. 2020). Animal studies suggest that protocatechuic acid may have therapeutic potential in models of neurodegenerative diseases, including chemotherapeutic drug‐induced brain injury in rats, and can effectively mitigate oxidative stress‐induced injury (Salama et al. 2022). Furthermore, protocatechuic acid has demonstrated significant nephroprotective effects in an acute kidney injury (AKI) mouse model by modulating the inflammatory response and the oxidative stress cascade pathway (Salama et al. 2023). These findings suggest that Lanmaoa asiatica may possess potential pharmacological activities, particularly in antioxidant, anti‐inflammatory, and neuroprotective capacities.

In the present study, we observed that key energy metabolism‐related substances, such as adenosine‐5′‐triphosphate (ATP) and adenosine‐5′‐diphosphate (ADP), were significantly upregulated in the plasma of patients with Lanmaoa asiatica intoxication. Furthermore, KEGG pathway analysis revealed significant alterations in the oxidative phosphorylation pathway, which is crucial for maintaining mitochondrial function and cellular energy metabolism (Zhang et al. 2024). These changes indicate abnormal fluctuations in metabolites closely associated with energy metabolism, suggesting potential impairment of mitochondrial function in these patients.

Mitochondria are central to cellular energy metabolism. Abnormal mitochondrial function is often accompanied by decreased oxidative phosphorylation efficiency, leading to a compensatory enhancement of the glycolytic pathway to maintain ATP supply. This compensatory response may result in a transient increase in ATP levels. The significant upregulation of ATP and ADP levels observed in this study may be associated with the heightened cellular repair demands under toxic stress, which drives the acceleration of ATP synthesis. Furthermore, the mushroom toxin α‐amanitin has been shown to inhibit the activity of the mitochondrial electron transport chain complex, resulting in a limited cellular energy supply and subsequent multi‐organ damage (Chen et al. 2020, 2021). Similarly, aconitine toxins, which are predominantly neurotoxic, have been demonstrated to cause structural damage to mitochondria (Li, Zhang, et al. 2023).

Additionally, toxicity‐induced stress may further inhibit ATPase activity, leading to abnormal accumulation of intracellular ATP levels and exacerbating energy metabolism disorders. The results of this study suggest that Lanmaoa asiatica poisoning may disrupt normal physiological functions by affecting the oxidative phosphorylation pathway and triggering energy metabolism disorders. Notably, brain tissue is susceptible to energy metabolism disruptions; thus, patients with Lanmaoa asiatica intoxication often exhibit significant neuropsychiatric symptoms. This finding provides a new research direction for further exploring the toxicity mechanisms of *Lanmaoa asiatica* and its impact on energy metabolism.

In this study, we systematically investigated the plasma metabolic profiles of patients suffering from *poisoning by Lanmaoa asiatica* using untargeted metabolomics analysis for the first time, thereby addressing a significant research gap in this field. The findings not only provide new insights into the toxicity mechanisms associated with *Lanmaoa asiatica* poisoning but also identify potential intervention targets that hold substantial scientific and clinical value. However, this study has several limitations. First, the relatively small sample size and the constraints of the study design may affect the generalizability of the findings. Also, the heterogeneity of clinical phenotype groupings may affect the interpretation of the results. Consequently, future research should aim to increase the sample size and conduct multicenter studies further to validate our findings with more comprehensive clinical data. Second, while this study primarily utilized metabolomics analysis, the specific mechanisms of the metabolic pathways have yet to be experimentally verified. Future investigations should integrate proteomics and transcriptomics data, and utilize animal models or cellular experiments further to examine the effects of toxins on energy metabolism. Additionally, intervention strategies, such as nicotinamide and coenzyme Q10 supplementation, should be designed to target the oxidative phosphorylation pathway, with their clinical efficacy evaluated in toxicity models. Furthermore, by measuring changes in the activities of key enzymes, such as ATP synthase and adenylate kinase, we can conduct a more in‐depth analysis of the molecular mechanisms of the toxin, providing a more accurate scientific basis for the prevention and treatment of *poisoning by Lanmaoa asiatica*.

## 5 Conclusions

In summary, the present study systematically compared the plasma metabolic profiles of patients intoxicated with *Lanmaoa asiatica* to those of healthy controls through non‐targeted metabolomic analysis. The results indicated that the overall metabolic patterns of intoxicated patients differed significantly from those of the normal population. *Lanmaoa asiatica* intoxication primarily affects energy metabolism and amino acid metabolism pathways, leading to significant metabolic disorders. Notably, 5‐methoxytryptophan and protocatechuic acid, which are metabolites of *Lanmaoa asiatica*, exhibit important pharmacological activities, warranting further investigation into their biological functions and potential clinical applications. Additionally, this study identified potential biomarkers related to the oxidative phosphorylation pathway, providing a theoretical basis for further clinical validation studies and establishing a foundation for analyzing poisoning mechanisms and exploring intervention strategies. However, this study has certain limitations. The relatively small sample size may restrict the statistical power and generalizability of the findings. Moreover, the inherent challenges of metabolite identification in untargeted metabolomics may affect the accuracy of metabolite annotation and pathway interpretation. Future studies with larger, clinically stratified cohorts and integrated, targeted metabolomic validation are needed to refine the mechanistic understanding further and identify reliable intervention targets.

## Author Contributions

**Ruanxian Dai:** funding acquisition (supporting), writing – original draft (equal). **Zhantao Duan:** data curation (equal). **Bin Han:** project administration (equal). **Yating Peng:** data curation (equal), investigation (equal). **Lan Zhu:** data curation (equal). **Yuan Shen:** writing – review and editing (equal). **Qiang Meng:** conceptualization (lead), writing – review and editing (equal).

## Ethics Statement

The study received approval from the Medical Ethics Committee of the First People's Hospital of Yunnan Province (Approval No. KHLL2023‐KY064) and adhered strictly to the ethical principles outlined in the Declaration of Helsinki. All tests were conducted after obtaining informed consent from the subjects or their legal guardians.

## Conflicts of Interest

The authors declare no conflicts of interest.

## Supporting information


**Figure S1.** The overall sample injection order and the PC1 control chart are presented. The horizontal axis represents the sample order, while the vertical axis illustrates the PC1 values. The yellow and red lines delineate the ranges of plus or minus 2 and 3 standard deviations, respectively. The green dots indicate the quality control (QC) samples, whereas the black dots represent the test samples.

**Table S1.** Some differential metabolites between the patient group and the healthy control group.

## Data Availability

The data that support the findings of this study are available from the corresponding author upon reasonable request.Author elects to not share data Research data are not shared.

## References

[fsn370583-bib-0001] Chen, X. , B. Shao , C. Yu , et al. 2020. “The Cyclopeptide <Alpha>−Amatoxin Induced Hepatic Injury via the Mitochondrial Apoptotic Pathway Associated With Oxidative Stress.” Peptides 129: 170314. 32387737 10.1016/j.peptides.2020.170314

[fsn370583-bib-0002] Chen, X. , B. Shao , C. Yu , et al. 2021. “Energy Disorders Caused by Mitochondrial Dysfunction Contribute to α‐Amatoxin‐Induced Liver Function Damage and Liver Failure.” Toxicology Letters 336: 68–79. 33098907 10.1016/j.toxlet.2020.10.003

[fsn370583-bib-0003] Cheng, H. , J. Zhao , J. Zhang , et al. 2023. “Attribution Analysis of Household Foodborne Disease Outbreaks in China, 2010–2020.” Foodborne Pathogens and Disease 20, no. 8: 358–367. 37506344 10.1089/fpd.2022.0070

[fsn370583-bib-0004] Chou, H. C. , and H. L. Chan . 2014. “5‐Methoxytryptophan‐Dependent Protection of Cardiomyocytes From Heart Ischemia Reperfusion Injury.” Archives of Biochemistry and Biophysics 543: 15–22. 24384558 10.1016/j.abb.2013.12.014

[fsn370583-bib-0005] Dai, R. , Z. Duan , J. Yang , et al. 2024. “Neuropsychiatric Symptoms Following the Consumption of Lanmaoa Asiatica, a Poisonous Mushroom Native to Yunnan.” Hong Kong Journal of Emergency Medicine 31, no. 6: 299–303.

[fsn370583-bib-0006] Diaz, J. H. 2021. “Nephrotoxic Mushroom Poisoning: Global Epidemiology, Clinical Manifestations, and Management.” Wilderness & Environmental Medicine 32, no. 4: 537–544. 34629291 10.1016/j.wem.2021.09.002

[fsn370583-bib-0007] Kermeoğlu, F. , U. Aksoy , A. Sebai , et al. 2021. “Anti‐Inflammatory Effects of Melatonin and 5‐Methoxytryptophol on Lipopolysaccharide‐Induced Acute Pulpitis in Rats.” BioMed Research International 2021: 8884041. 33628825 10.1155/2021/8884041 PMC7895566

[fsn370583-bib-0008] Kupnicka, P. , K. Kojder , E. Metryka , et al. 2020. “Morphine‐Element Interactions—The Influence of Selected Chemical Elements on Neural Pathways Associated With Addiction.” Journal of Trace Elements in Medicine and Biology 60: 126495. 32179426 10.1016/j.jtemb.2020.126495

[fsn370583-bib-0009] Li, H. , M. B. Lockwood , J. M. Schlaeger , T. Liu , O. C. Danciu , and A. Z. Doorenbos . 2023. “Tryptophan and Kynurenine Pathway Metabolites and Psychoneurological Symptoms Among Breast Cancer Survivors.” Pain Management Nursing 24, no. 1: 52–59. 36229337 10.1016/j.pmn.2022.09.002 PMC9925397

[fsn370583-bib-0010] Li, H. , Y. Zhang , H. Zhang , et al. 2023. “Mushroom Poisoning Outbreaks—China, 2022.” China CDC Weekly 5, no. 3: 45–50. 36776462 10.46234/ccdcw2023.009 PMC9902756

[fsn370583-bib-0011] Li, H. J. , Y. Z. Zhang , Z. T. Liu , F. S. Zheng , B. Zhao , and G. Wu . 2022. “Species Diversity of Toxic Mushrooms in Mushroom Poisoning Incidents in Yunnan.” [In Chinese.] Mycosystema 41, no. 9: 1416–1429.

[fsn370583-bib-0012] Li, Q. , F. Peng , X. Yan , et al. 2023. “Inhibition of SLC7A11‐GPX4 Signal Pathway Is Involved in Aconitine‐Induced Ferroptosis In Vivo and In Vitro.” Journal of Ethnopharmacology 303: 116029. 36503029 10.1016/j.jep.2022.116029

[fsn370583-bib-0013] Li, W. , S. Pires , Z. Liu , et al. 2021. “Mushroom Poisoning Outbreaks—China, 2010–2020.” China CDC Weekly 3, no. 24: 518–522. 34594925 10.46234/ccdcw2021.134 PMC8393043

[fsn370583-bib-0014] Li, Y. , H. J. Li , Y. S. Fu , et al. 2023. “Epidemiological and Clinical Characteristics of Acute Lanmaoa Asiatica Poisoning.” [In Chinese.] Journal of Clinical Emergency 24, no. 5: 258–261.

[fsn370583-bib-0015] Liu, J. Y. , X. M. Yang , Y. Y. Chen , et al. 2023. “Analysis of Associated Substances and Major Pathways in the Fruiting Body Development of Lanmaoa Asiatica.” [In Chinese.] Microbiology China 50, no. 9: 4045–4062.

[fsn370583-bib-0016] Moss, M. J. , and R. G. Hendrickson . 2019. “Toxicity of Muscimol and Ibotenic Acid Containing Mushrooms Reported to a Regional Poison Control Center From 2002‐2016.” Clinical Toxicology 57, no. 2: 99–103. 30073844 10.1080/15563650.2018.1497169

[fsn370583-bib-0017] Ouzir, M. , N. Bouhaddou , H. Khalki , and N. Lakhdar‐Ghazal . 2013. “Physiological and Pharmacological Properties of 5‐Methoxytryptophol.” Expert Review of Endocrinology & Metabolism 8, no. 4: 355–364. 30736152 10.1586/17446651.2013.811866

[fsn370583-bib-0018] Salama, A. , R. Elgohary , M. M. Amin , and S. A. Elwahab . 2022. “Immunomodulatory Effect of Protocatechuic Acid on Cyclophosphamide Induced Brain Injury in Rat: Modulation of Inflammosomes NLRP3 and SIRT1.” European Journal of Pharmacology 932: 175217. 36007603 10.1016/j.ejphar.2022.175217

[fsn370583-bib-0019] Salama, A. A. A. , R. Elgohary , and M. I. Fahmy . 2023. “Protocatechuic Acid Ameliorates Lipopolysaccharide‐Induced Kidney Damage in Mice via Downregulation of TLR‐4‐Mediated IKBKB/NF‐κB and MAPK/Erk Signaling Pathways.” Journal of Applied Toxicology 43, no. 8: 1119–1129. 36807594 10.1002/jat.4447

[fsn370583-bib-0020] Şehirli, A. , and S. Sayıner . 2021. “Daylight Is Critical to Preserve 5‐Methoxytryptophol Levels in Suspected and Confirmed COVID‐19 Patients.” Medical Hypotheses 147: 110504. 33485026 10.1016/j.mehy.2021.110504 PMC7816952

[fsn370583-bib-0021] Somrithipol, S. , U. Pinruan , S. Sommai , P. Khamsuntorn , and J. J. Luangsa‐Ard . 2022. “Mushroom Poisoning in Thailand Between 2003 and 2017.” Mycoscience 63, no. 6: 267–273. 37089521 10.47371/mycosci.2022.08.003 PMC10042301

[fsn370583-bib-0022] Song, J. , Y. He , C. Luo , et al. 2020. “New Progress in the Pharmacology of Protocatechuic Acid: A Compound Ingested in Daily Foods and Herbs Frequently and Heavily.” Pharmacological Research 161: 105109. 32738494 10.1016/j.phrs.2020.105109

[fsn370583-bib-0023] Su, L. , J. Y. Su , D. Li , et al. 2018. “Effects of Serum Containing Lanmaoa Asiatica Extract on Spleen Lymphocytes in Mice.” [In Chinese.] Journal of Edible Fungi 25, no. 2: 113–120.

[fsn370583-bib-0024] Tang, J. , R. Xu , H. Ma , et al. 2024. “Two Alkenyl Phenol Derivatives From the Fungus Pestalotiopsis Clavata JSQ 12 Isolated From the Mushroom Lanmaoa Asiatica.” Natural Product Research: 1–5. 10.1080/14786419.2024.2367012. 38867712

[fsn370583-bib-0025] Wang, Y. , Y. Lüli , X. Li , Z. L. Yang , and H. Luo . 2024. “Pulvinic Acid Derivative Pigments in Lanmaoa Asiatica and *L. macrocarpa* .” Chemistry & Biodiversity 21, no. 5: e202301996. 38509847 10.1002/cbdv.202301996

[fsn370583-bib-0026] Wang, Y. F. , Y. J. Hsu , H. F. Wu , et al. 2016. “Endothelium‐Derived 5‐Methoxytryptophan Is a Circulating Anti‐Inflammatory Molecule That Blocks Systemic Inflammation.” Circulation Research 119, no. 2: 222–236. 27151398 10.1161/CIRCRESAHA.116.308559

[fsn370583-bib-0027] Wu, K. K. 2021. “Control of Tissue Fibrosis by 5‐Methoxytryptophan, an Innate Anti‐Inflammatory Metabolite.” Frontiers in Pharmacology 12, no. 11: 759199. 34858185 10.3389/fphar.2021.759199 PMC8632247

[fsn370583-bib-0028] Wu, K. K. , H. H. Cheng , and T. C. Chang . 2014. “5‐Methoxyindole Metabolites of L‐Tryptophan: Control of COX‐2 Expression, Inflammation and Tumorigenesis.” Journal of Biomedical Science 21, no. 1: 17. 24589238 10.1186/1423-0127-21-17 PMC3975872

[fsn370583-bib-0029] Wu, K. K. , C. C. Kuo , S. F. Yet , C. M. Lee , and J. Y. Liou . 2020. “5‐Methoxytryptophan: An Arsenal Against Vascular Injury and Inflammation.” Journal of Biomedical Science 27, no. 1: 79. 32635910 10.1186/s12929-020-00671-w PMC7341587

[fsn370583-bib-0030] Yang, N. , S. Zhang , P. Zhou , et al. 2022. “Analysis of Volatile Flavor Substances in the Enzymatic Hydrolysate of Lanmaoa Asiatica Mushroom and Its Maillard Reaction Products Based on E‐Nose and GC‐IMS.” Food 11, no. 24: 4056. 10.3390/foods11244056 PMC9778328 36553801

[fsn370583-bib-0031] Zhang, X. , H. Gong , Y. Zhao , et al. 2024. “Bisphenol S Impairs Mitochondrial Function by Targeting Myo19/Oxidative Phosphorylation Pathway Contributing to Axonal and Dendritic Injury.” Environment International 186: 108643. 38615544 10.1016/j.envint.2024.108643

[fsn370583-bib-0032] Zheng, C. , S. Lv , J. Ye , et al. 2023. “Metabolomic Insights Into the Mechanisms of Ganoderic Acid: Protection Against α‐Amanitin‐Induced Liver Injury.” Metabolites 13, no. 11: 1164. 37999259 10.3390/metabo13111164 PMC10672867

[fsn370583-bib-0033] Zheng, F. , X. Zhao , Z. Zeng , et al. 2020. “Development of a Plasma Pseudotargeted Metabolomics Method Based on Ultra‐High‐Performance Liquid Chromatography‐Mass Spectrometry.” Nature Protocols 15, no. 8: 2519–2537. 32581297 10.1038/s41596-020-0341-5

