# Supplementary figures and images for: Untargeted Metabolomic Analysis Using UPLC–MS/MS Reveals Metabolic Changes Associated With Lanmaoa asiatica Poisoning
Source: Food Sci Nutr. 2025 Jul 8;13(7):e70583. doi: 10.1002/fsn3.70583 (PMC12237617; doi:10.1002/fsn3.70583)

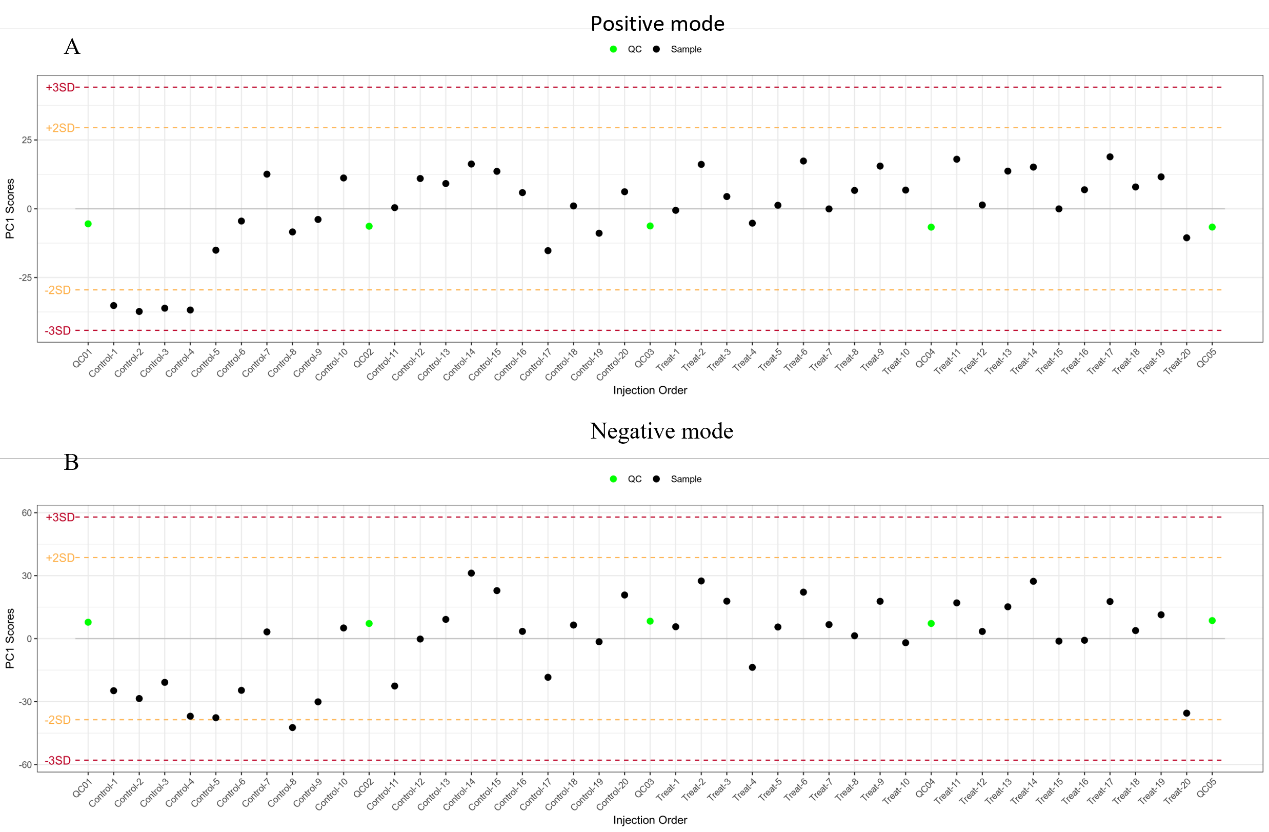

Supplement: Supplementary file 1 — Figure S1. The overall sample injection order and the PC1 control chart are presented. The horizontal axis represents the sample order, while the vertical axis illustrates the PC1 values. The yellow and red lines delineate the ranges of plus or minus 2 and 3 standard deviations, respectively. The green dots indicate the quality control (QC) samples, whereas the black dots represent the test samples. [file FSN3-13-e70583-s001.docx]
