# Supplementary material for: Untargeted Metabolomic Analysis Using UPLC–MS/MS Reveals Metabolic Changes Associated With Lanmaoa asiatica Poisoning
Source: Food Sci Nutr. 2025 Jul 8;13(7):e70583. doi: 10.1002/fsn3.70583 (PMC12237617; doi:10.1002/fsn3.70583)
Supplement: Supplementary file 2 — Table S1. Some differential metabolites between the patient group and the healthy control group. [file FSN3-13-e70583-s002.docx]

Table S1 Some differential metabolites between the patient group and the healthy control group

| Compounds name | VIP | P-value | Log2FC | Trend |
| --- | --- | --- | --- | --- |
| 2-ammonio-3-(5-hydroxy-1H-indol-3-yl)propanoate | 2.301851 | 0.001031394 | 5.744884 | up |
| Propionic acid, 2-amino-3-ureido- | 2.799009 | 6.80738E-06 | 5.712127 | up |
| alpha-CYANO-3-HYDROXYCINNAMIC ACID | 2.259193 | 0.001700338 | 5.173719 | up |
| 5-Methoxytryptophol | 2.940652 | 0.00032899 | 4.901358 | up |
| Rubiadin | 2.751184 | 2.71969E-05 | 4.582678 | up |
| Protocatechuic acid | 2.569546 | 0.000545936 | 4.262486 | up |
| Carnitine | 2.847889 | 1.49534E-05 | 4.246272 | up |
| Inosinic acid | 2.506527 | 1.6865E-05 | 4.00856 | up |
| 4-Hydroxy-5-(3,4,5-trihydroxyphenyl)pentanoic acid | 2.838284 | 4.43051E-07 | 3.873564 | up |
| 1-Acetylproline | 2.414625 | 7.12549E-06 | 3.752232 | up |
| Adenosine-5'-triphosphate | 2.364649 | 0.000109388 | 3.381421 | up |
| Adenosine 5'-Diphosphate | 2.488403 | 4.56964E-06 | 3.296451 | up |
| Zuclopenthixol | 1.211769 | 0.035047547 | 3.233029 | up |
| S-methyl-5-thio-alpha-D-ribose 1-phosphate | 2.200339 | 0.000386759 | 2.98135 | up |
| N,N-Dimethyl-L-Valine | 2.362761 | 0.003161261 | 2.920527 | up |
| Docusate | 2.307143 | 0.000988319 | -2.90111 | down |
| Enantio-PAF C-16 | 1.08555 | 0.03769425 | -3.34742 | down |
| 1-(1Z-hexadecenyl)-sn-glycero-3-phosphocholine | 1.222104 | 0.049893147 | -3.7227 | down |
| Jubanine C | 1.764938 | 0.015034716 | -4.10941 | down |
| 3-O-Protocatechuoylceanothic acid | 1.80334 | 0.012447198 | -5.30835 | down |
